# Flavin fluorescence lifetime and autofluorescence optical redox ratio for improved visualization and classification of brain tumors

**DOI:** 10.3389/fonc.2023.1105648

**Published:** 2023-02-20

**Authors:** David Reichert, Lisa I. Wadiura, Mikael T. Erkkilae, Johanna Gesperger, Alexandra Lang, Thomas Roetzer-Pejrimovsky, Jessica Makolli, Adelheid Woehrer, Marco Wilzbach, Christoph Hauger, Barbara Kiesel, Marco Andreana, Angelika Unterhuber, Wolfgang Drexler, Georg Widhalm, Rainer A. Leitgeb

**Affiliations:** ^1^ Center for Medical Physics and Biomedical Engineering, Medical University of Vienna, Vienna, Austria; ^2^ Christian Doppler Laboratory for Innovative Optical Imaging and its Translation to Medicine (OPTRAMED), Medical University of Vienna, Vienna, Austria; ^3^ Department of Neurosurgery, General Hospital and Medical University of Vienna, Vienna, Austria; ^4^ Division of Neuropathology and Neurochemistry, Department of Neurology, Medical University of Vienna, Vienna, Austria; ^5^ Advanced Development Microsurgery, Carl Zeiss Meditec AG, Oberkochen, Germany

**Keywords:** fluorescence guided surgery, fluorescence lifetime imaging, fluorescence spectroscopy, optical redox ratio, flavin mononucleotide

## Abstract

**Purpose:**

Modern techniques for improved tumor visualization have the aim to maximize the extent of resection during brain tumor surgery and thus improve patient prognosis. Optical imaging of autofluorescence is a powerful and non-invasive tool to monitor metabolic changes and transformation in brain tumors. Cellular redox ratios can be retrieved from fluorescence emitted by the coenzymes reduced nicotinamide adenine dinucleotide (phosphate) (NAD(P)H) and flavin adenine dinucleotide (FAD). Recent studies point out that the influence of flavin mononucleotide (FMN) has been underestimated.

**Experimental design:**

Fluorescence lifetime imaging and fluorescence spectroscopy were performed through a modified surgical microscope. We acquired 361 flavin fluorescence lifetime (500-580 nm) and fluorescence spectra (430-740 nm) data points on freshly excised different brain tumors: low-grade gliomas (N=17), high-grade gliomas (N=42), meningiomas (N=23), metastases (N=26) and specimens from the non-tumorous brain (N=3).

**Results:**

Protein-bound FMN fluorescence in brain tumors did increase with a shift toward a more glycolytic metabolism (*R=-0.87*). This increased the average flavin fluorescence lifetime in tumor entities with respect to the non-tumorous brain. Further, these metrics were characteristic for the different tumor entities and showed promise for machine learning based brain tumor classification.

**Conclusions:**

Our results shed light on FMN fluorescence in metabolic imaging and outline the potential for supporting the neurosurgeon in visualizing and classifying brain tumor tissue during surgery.

## 1 Introduction

Complete resection with preservation of neurological function is the neurosurgical aim in most brain tumors to improve patient prognosis (1–6). In this respect, the importance of the extent of resection (EOR) has been demonstrated especially for low-grade gliomas (LGG) (1, 2), high-grade gliomas (HGG) (2, 7, 8), meningiomas (MNG) (5) and brain metastases (MET) (6). In the last years, modern techniques for optical intraoperative tumor visualization with fluorescence using exogenous dyes such as 5-aminolevulinic acid (5-ALA) have been introduced to the neurosurgical field supporting the surgeon in maximizing the EOR (7, 9–14). With the aim to further optimize these techniques, we developed a surgical microscope with the capability to measure the fluorescence lifetime of fluorophores, which is the average intramolecular time delay between excitation of a fluorophore and the emission of fluorescence (15, 16). Additionally, a spectrometer integrated into the microscope allowed for capturing the fluorescence emitted by the tissue across the visible spectrum. The combination of these techniques was used in this work to analyse tissue autofluorescence in brain tumors.

Endogenous fluorophores provide an alternate source of fluorescence visualization without the need of systemic dye administration. In particular, fluorescence emitted by the coenzymes reduced nicotinamide adenine dinucleotide (phosphate) (NAD(P)H) and flavin adenine dinucleotide (FAD) are commonly used to measure the cellular metabolism of tumors (17, 18). A higher fraction of glycolytic catabolism with respect to oxidative phosphorylation (OXPHOS) despite the availability of oxygen in tumor cells was first described by Warburg et al. (19, 20). Initially, this was thought to be due to defects in mitochondrial respiration (19–21). Luengo et al. recently presented an updated model showing that proliferative cells can reach a state where the demand for NAD+ to fuel oxidation reactions exceeds the demand for adenosine triphosphate (ATP) (21). This results in an increase of free NAD(P)H fluorescence, which is localized in the cytosol during glycolytic processes. Contrarily, FAD fluorescence increases during OXPHOS as it is oxidized from non-fluorescent FADH_2_ in the electron transport chain (22). The ratio of the emitted fluorescence of FAD and NAD(P)H can be expressed as optical redox ratio and allows for non-invasive monitoring of the cellular redox state (17, 18, 23). In addition, fluorescence lifetime induced redox ratios (FLIRR) have been proposed to correlate the relative amount of bound FAD and NAD(P)H (24). The intensity based redox ratio as well as FLIRR have demonstrated promising results in a variety of *in-vitro* to *in-vivo* studies to contrast alterations in tumors with respect to non-tumorous tissue (22, 25–29). The role of flavin fluorescence in metabolic imaging, however, is complicated and not widely understood. Recent work outlined that the contributions from protein-bound flavin mononucleotide (FMN) had been underestimated (25).

In this study, we investigated the use of flavin fluorescence for brain tumor detection and classification by shedding light on the dynamics of flavin fluorescence associated with metabolic alterations in brain tumor tissue. To this end, we combined the flavin fluorescence lifetime with fluorescence spectra acquired *ex-vivo* on freshly collected specimens of different brain tumor entities with our multimodal surgical microscope (15, 16).

## 2 Materials and methods

### 2.1 Imaging system

Frequency-domain fluorescence lifetime imaging (FD-FLIM) and spectroscopic measurements were performed with a multimodal surgical microscope as recently described (15, 16). We acquired FD-FLIM images *via* raster-scanning at a working distance of 200 mm. The field of view was 6.5 x 6.5 mm^2^. Fluorescence was filtered in the range of flavin emission from 500 nm to 580 nm (BrightLine HC Semrock, Rochester, New York) for FD-FLIM. Fluorescence spectra were acquired on spatially correlated areas (0.6 x 0.6 mm^2^) from 430 nm to 740 nm. Our system employed a diode laser (phoxX-405, Omicron Laserage, Rodgau, Germany) at 405 nm excitation with 6 mW of laser power at the sample plane. Post-processing routines for reconstructing the fluorescence lifetime and intensity images as well as for processing the acquired spectra were implemented in Python (RRID : SCR_008394) as described previously (16).

### 2.2 Metrics for optical metabolic imaging

We defined the optical redox ratio RR (17, 18) as


$$RR=\;\frac{FAD}{NAD\left(P\right)H+FAD}\;,$$

and obtained the NAD(P)H and FAD fluorescence when integrating the spectra from 430 nm to 475 nm and 520 nm to 600 nm, respectively. The choice of these bands was based on validations by Cao et al. for minimized leakage when exciting with a single excitation wavelength (30). It is of note that different conventions of the redox ratio exist in the literature (31). Further, we defined a ratio $R_{flavin}=\;\frac{I_{495}}{I_{530}}$ between the fluorescence intensities at the peaks at 495 nm (32, 33) and 530 nm (34), which reflects the relative contributions of protein-bound FMN and FAD fluorescence, respectively. Furthermore, the redox ratio and *R_flavin_* were correlated with the flavin fluorescence lifetime acquired on the same area.

### 2.3 Patient cohort

In this study, we included the following ex-vivo patient data. Note that for some patients multiple specimens were available for analysis: MET (26 specimens/22 patients), LGG (17/11), HGG (42/33) and MNG (23/22). Furthermore, 3 non-tumorous control (CTL) specimens from 2 patients primarily collected during the approach to deep-seated tumors were available for analysis. The age range and male to female ratio for the cohort are shown in **Table 1**. Based on the obtained flavin fluorescence lifetime image and the multiple fluorescence spectra the following number of data points for the combined analysis of fluorescence lifetime and spectra were included: 93 MET, 71 LGG, 117 HGG, 64 MNG and 16 CTL.

**Table 1:** Clinical data for the patient cohort; female (f), male (m).

	Control		Low-grade glioma		High-grade glioma		Meningioma		Metastases	
**Gender**	f	m	f	m	f	m	f	m	f	m
**Number of patients**	2	–	8	3	16	17	14	8	15	7
**Age (years)**	68±3	–	35±7	36±12	53±16	58±12	64±10	63±14	60±10	61±8

All tumor samples were collected during routine neurosurgical resection at the Department of Neurosurgery, Medical University Vienna. The specimens were imaged usually within 1 hour after resection and preserved in artificial cerebrospinal fluid (Landesapotheke Salzburg, 19C11S02). A flavin fluorescence lifetime image and multiple fluorescence spectra were obtained for each specimen. Histopathological diagnosis was performed according to the valid World Health Organization (WHO) classification at the time of diagnosis (35). During imaging and post-processing, experimenters were blinded to the subjects’ diagnosis. This study was approved by the local ethics committee of the Medical University of Vienna (*EC no.: 419/2008, amendment*) and informed consent was given by all patients.

### 2.4 Statistical analysis

Data were visualized with violin plots and statistical inference between the tumor entities and a control group was checked with a non-parametric Mann-Whitney-U test. Differences in the distributions among subgroups were ruled out with the Kolmogorov-Smirnov-test after data normalization. We considered differences between groups to be significant if p< 0.05, with the alternate hypotheses stating that (1) the median flavin fluorescence lifetime was significantly greater, (2) the redox ratio was significantly lower and (3) *R_flavin_* was significantly greater for each of the tumor entities with respect to the non-pathological control group.

### 2.5 Machine learning

Flavin fluorescence lifetime, the redox ratio, and *R_flavin_* were then used as input features for tumor classification with machine learning models. We set up a pipeline to evaluate the performance of various models (LinearDiscriminantAnalysis, LogisticRegression, KNeighborsClassifier, SupportVectorClassification, GradientBoostingClassifier, RandomForestClassifier, ExtraTreesClassifier) on our data. Using the F-score as a metric, models were evaluated with stratified 5-fold cross-validation which was repeated 50 times (train/test split 80/20). For the best model we calculated the multi-class sensitivity across the validation cases of all folds, which essentially gave use the average model performance for all 250 validations with the randomly varied folds of our data. We make the case that this provides a much more conservative and reliable estimate of the actual model performance when compared to testing model performance with a single test-set of data put aside before the training. This is especially the case for the rather small datasets common to biomedical imaging. Within each validation, we employed the synthetic minority oversampling technique to augment training data within the minority groups to the size of the majority group.

### 2.6 Data availability

The data generated in this study are available upon request to the corresponding author.

## 3 Results

### 3.1 Flavin fluorescence and optical redox ratio for brain tumor detection

**Figure 1:**
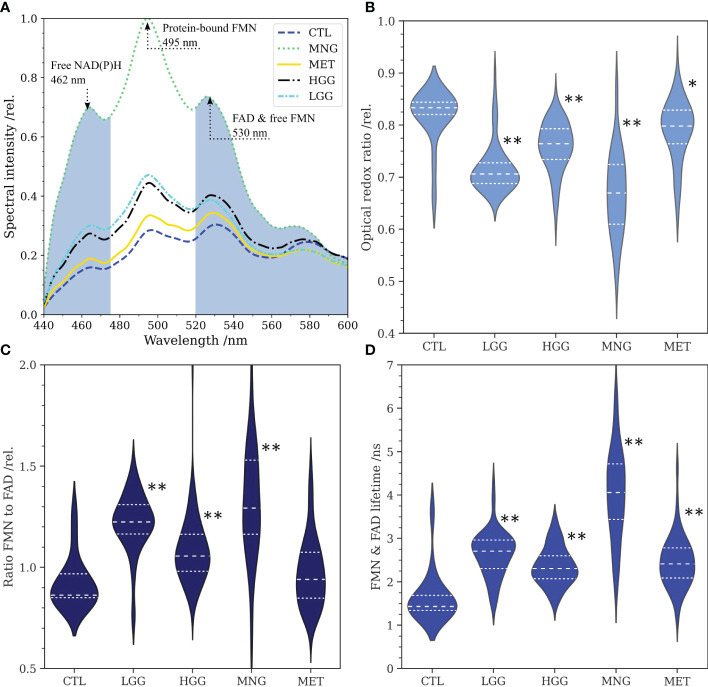
**(A)**:Average fluorescence spectra of low- and high grade gliomas (LGG, n=71, HGG, n=117), meningiomas (MNG, n=64), brain metastases (MET, n=93) and non-tumorous brain (CTL, n=16) for excitation at 405 nm. The spectral bands for integration of NAD(P)H and FAD fluorescence are colored in grey. Note the peaks at about 462 nm (free NAD(P)H), 495 nm (FMN) and 530 nm (FAD, side peak of FMN). **(B)**: Optical redox ratios obtained from the integration of NAD(P)H and FAD fluorescence in spectra according to equation 1. **(C)**: Ratio of the peaks at 495 nm and 530 nm reflecting the ratio of protein-bound FMN to FAD. **(D)**: Flavin fluorescence lifetime for the spectral range from 500 nm to 580 nm. For **(B–D)**, statistical significant differences of the tumor entities to the control group are indicated through asterisks (* p<0.005, ** p<1e-5).

Since a higher fraction of glycolytic catabolism implies that less FMN is reduced to FMNH_2_ in the mitochondria, we hypothesized that (1) protein-bound FMN accumulates with increasing glycolytic activity in brain tumors and (2) this leads to increased flavin fluorescence lifetimes dominated by the long lifetime component of FMN. To this end, we acquired flavin fluorescence lifetime data and extracted optical redox ratios as well as the protein-bound FMN to FAD ratio *R_flavin_* from fluorescence spectra. **Figure 1A** shows the average fluorescence spectra for the different groups. According to our data, fluorescence of free NAD(P)H peaking at 462 nm (34) was increased for all tumor groups with respect to the CTL group. In detail, MNG showed the strongest increase (rel. spectral intensity = 0.69) followed by LGG (0.29) and HGG (0.27). In contrast, MET (0.18) showed only a small increase compared to CTL (0.15). A strong increase in the same order (MNG: 1.00, LGG: 0.47, HGG: 0.44, MET: 0.33, CTL: 0.28) was also found for the peak at 495 nm, corresponding to the main emission peak of protein-bound FMN. The same result was observed for the peak at about 530 nm, where a side peak of FMN and the main emission peak of FAD is expected (MNG: 0.72, LGG: 0.38, HGG: 0.40, MET: 0.34, CTL: 0.30). **Figure 1B-D** illustrate the distribution of the redox ratio, R*_flavin_* and the flavin lifetime for all groups. The redox ratio was significantly reduced for LGG (p=7.765e-7), HGG (p=1.045e-6), MNG (p=1.270e-7) and MET (p=4.263e-3) with respect to CTL tissue, implying a higher fraction of glycolytic catabolism in tumorous tissue. At the same time, *R_flavin_* was significantly increased for all tumor groups **Figure 1C**); LGG (p=2.018e-7), HGG (p=6.879e-6), MNG (p=1.063e-7) and MET (p=1.074e-1). The accumulation of FMN with increased glycolytic activity led to an increase of the average flavin fluorescence lifetime, which also was significantly higher for LGG (p=1.426e-7), HGG (p=1.470e-7), MNG (p=1.964e-9), but not significant for MET (p=1.074e-1) (**Figure 1D**). For detailed information on data shown in the violin plots in B-D please refer to **Table 2**.

**Table 2:** Descriptive and inferential data for the optical redox ratio, the ratio of FMN to FAD and the flavin fluorescence lifetime from **Figure 1**.

	Control	Low-grade glioma	High-grade glioma	Meningioma	Metastases
Optical redox ratio					
**Median**	0.83	0.71	0.76	0.67	0.8
**0.25 quartile**	0.82	0.69	0.73	0.61	0.76
**0.75 quartile**	0.84	0.73	0.79	0.72	0.83
**p-value**	–	7.765e-7	1.045e-6	1.270e-7	4.263e-3
Ratio protein-bound FMN to FAD *R_flavin_*					
**Median**	0.86	1.22	1.06	1.29	0.94
**0.25 quartile**	0.85	1.17	0.98	1.16	0.85
**0.75 quartile**	0.97	1.31	1.16	1.53	1.08
**p-value**	–	2.018e-7	6.879e-6	1.063e-7	1.074e-1
Flavin fluorescence lifetime 500 nm - 580 nm					
**Median**	1.43	2.71	2.31	4.06	2.41
**0.25 quartile**	1.34	2.31	2.07	3.44	2.09
**0.75 quartile**	1.69	2.97	2.6	4.72	2.78
**p-value**	–	1.426e-7	1.470e-7	1.964e-9	5.167e-7

### 3.2 Flavin fluorescence and optical redox ratio for the identification of tumor entities

**Figure 2:**
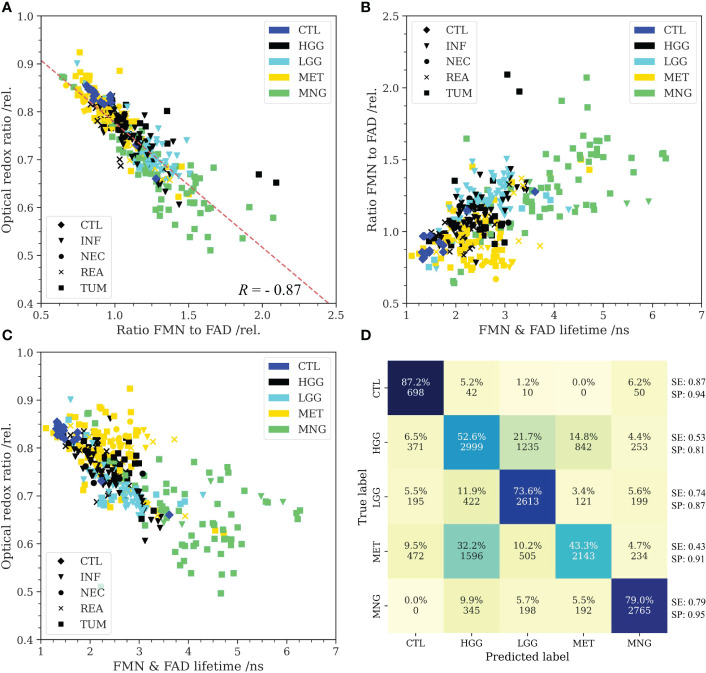
**(A)**: Higher protein-bound FMN fluorescence with respect to FAD was found to correlate with a reduced optical redox ratio (R = -0.87). All tumor entities predominantly showed reduced redox ratios and higher FMN fluorescence than non-tumorous brain (control, CTL). Only metastases (MET) were found to be heterogeneous and partly overlapped with the non-tumorous brain cluster. **(B)**: The amount of FMN fluorescence plotted as a function of the flavin lifetime (500-580 nm) showed characteristic clusters for the different groups. While MET weren’t easily distinguishable from the CTL group in the redox ratio, flavin lifetime was predominantly increased beyond the lifetimes in non-tumorous brain. **(C)**: Redox ratio plotted as a function of the flavin lifetime implied that the predominant metabolic strategy was characteristic for the different tumor entities. Note the high flavin lifetimes of MNG. Markers further indicate the predominant histopathological tissue classification for **(A-C)** (INF: tumor infiltrated brain, NEC: necrosis, REA: reactive tissue, TUM: core tumor). **(D)**: Confusion matrix for classification of the different groups with a support vector machine. Input to the model were the optical redox ratio, the FMN/FAD ratio and the flavin lifetime. Autofluorescence was highly characteristic for CTL and meningiomas (MNG). MET were partly misclassified as high-grade gliomas (HGG) implying similarities with respect to the predominant metabolic strategy. Further, classification uncertainty existed between low-grade gliomas (LGG) and HGG. Multi-class sensitivity (SE) and specificity (SP) are given at the end of each row.

We further hypothesized that the combined information of flavin fluorescence and the optical redox ratio reflects metabolic characteristics and thereby allows classification of the brain tumor entities investigated in this study. **Figure 2A** shows *R_flavin_* as a function of the optical redox ratio. Fitting a linear regression model (*R = -0.87*) emphasized that an increased fraction of glycolytic catabolism (reduced redox ratio) led to an increase in protein-bound FMN fluorescence with respect to FAD. All tumor entities predominantly showed reduced redox ratios and higher FMN fluorescence compared to samples from CTL. Within the MET group, a subgroup overlapped with the CTL group. When plotting *R_flavin_* (**Figure 2B**) and the redox ratio (**Figure 2C**) as a function of the flavin fluorescence lifetime, characteristic clusters could be observed for each tumor entity. We then fitted our data with a support vector machine. **Figure 2D** shows the respective confusion matrix with the multi-class sensitivity (SE) and specificity (SP) being depicted at the end of each row. The confusion matrix shows both the detection probability for each class and the total number of prediction for all 250 validation runs. The three features considered allowed for detecting non-tumorous brain with a SE and SP against all other classes of 87% and 94%, respectively. HGG were classified with a SE of 53% and SP of 81% and were falsely classified as LGG in 22% and MET in 15% of cases. LGG were classified with a SE of 74% and SP of 87% and falsely classified as HGG in 12% of cases. SE and SP for MET were 43% and 91%, respectively. The unclear decision boarder between HGG and MET (32% false prediction as HGG) also becomes apparent from the overlap of subgroups in **Figure 2A-C**. Flavin autofluorescence and the optical redox ratio were found to be very characteristic for MNG, with a SE of 79% and SP of 95%.

### 3.3 Illustration of flavin fluorescence lifetime and spectra for selected clinical cases

**Figure 3:**
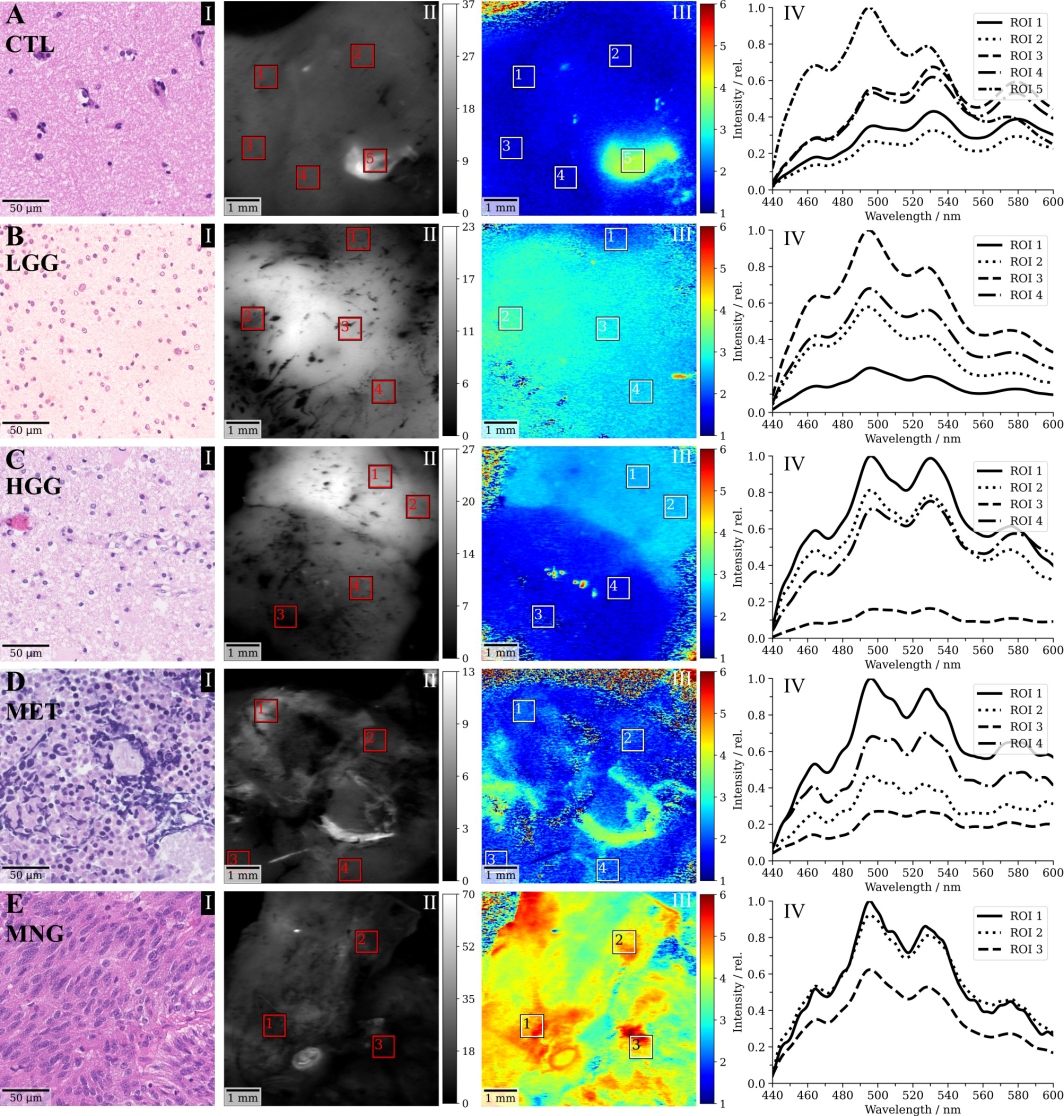
Selected clinical cases of **(A)** non-tumorous control (CTL) tissue, **(B)** a WHO grade II diffuse astrocytoma (low-grade, LGG), **(C)** a glioblasatoma (high-grade, HGG), **(D)** a bronchial carcinoma metastasized to the cerebellum and **(E)** a meningothelial meningioma (MNG). (I) shows a representative haematoxylin and eosin (H&E) stain of the respective sample, (II) the demodulated fluorescence intensity in mV_RMS_, (III) the flavin fluorescence lifetime in ns (500-580 nm) and (IV) the spectroscopic measurements corresponding to the regions indicated in III.

This section shows representative specimens to provide the reader with a better understanding for the data acquired and analyzed within this study. **Figure 3A** I shows the haematoxylin and eosin (H&E) stain of CNS tissue with only minor reactive changes for a non-tumorous specimen from the CTL group. The flavin lifetimes measured in the ROIs indicated through squares were 1.33 ns, 1.34 ns, 1.34 ns, 1.31 ns and 3.61 ns (in order from ROI 1 to 5). The high lifetimes in ROI 5 could indicate a focal tumor hotspot which was missed by the histopathological sampling. This was supported by the spectral measurements in **Figure 3A** IV. While ROI 1 to 4 show a characteristic increase from the peak of free NAD(P)H at 462 nm toward FAD at about 530 nm, free NAD(P)H and FMN at 495 nm were highly increased in ROI 5, indicating a shift to a more glycolytic metabolism. The respective redox ratios are 0.86, 0.85, 0.85, 0.84 and 0.66. **Figure 3B** I depicts CNS tissue with increased cellularity and infiltrating pleomorphic glial tumor cells of a diffuse astrocytoma WHO grade II, partially showing characteristics of transforming toward an anaplastic astrocytoma WHO grade III. Note the increased flavin lifetimes (2.10 ns, 3.14 ns, 2.83 ns, 2.67 ns) and low redox ratios (0.74, 0.67, 0.7, 0.72) with respect to the CTL sample in A, with the equivalent changes in fluorescence spectra shown in B-IV. **Figure 3C** shows tumor infiltrated CNS from a glioblastoma WHO grade IV with microvascular activation (C I). Here, regions of increased flavin lifetimes were found next to lifetimes slightly above the non-tumorous brain in A (2.51 ns, 2.54 ns, 1.56 ns, 1.46 ns), which was also reflected by the redox ratios (0.77, 0.76, 0.81, 0.82). Flavin lifetimes in a MET sample were 2.04 ns, 1.95 ns, 1.85 ns and 2.11 ns (**Figure 3D** III), with the H&E showing necrotic fragments of a bronchial carcinoma with small- to middle-sized cells and expression of neuroendocrine markers (**Figure 3D** I). The corresponding redox ratios were 0.79, 0.78, 0.8 and 0.78. It is of note, that an increase in flavin lifetime can be observed in parts of the sample where unfortunately no spectra were acquired. A meningothelial MNG WHO grade I is shown in **Figure 3E**, with a characteristic meningothelial appearance in the H&E (**Figure 3E** I). Flavin lifetimes were highly increased (4.79 ns, 4.43 ns, 4.65 ns) with redox ratios of 0.76, 0.75 and 0.73.

## 4 Discussion

### 4.1 Flavin fluorescence in optical redox imaging

While terminally differentiated cells rely on efficient ATP production through OXPHOS, tumorous transformation and increased cellular proliferation often entail higher fractions of aerobic glycolysis (36). This increases free cytosolic NAD(P)H and decreases FAD fluorescence which drives down the optical redox ratio (26, 36). Recent studies suggested that the role of FMN fluorescence has been underestimated in optical redox imaging (25). Protein-bound FMN has a quantum yield 10 times higher than FAD and fluorescence (spectral range 562/40 nm) and was found to be dominated by FMN in oral squamous carcinoma cell cultures (25). FAD in protein-bound compound has a short fluorescence lifetime component of about 0.3 ns and a long component of 2 ns to 3 ns for free FAD (22, 25, 37). During OXPHOS, the short lifetime contribution of FAD dominates, which is in contrast to the long fluorescence lifetime of protein-bound FMN in mitochondria of about 4.7 ns (38, 39). The increase of fluorescence at 495 nm in our data was highly comparable to the emission of FMN-binding fluorescent proteins (32, 33), that obtain their photophysical properties through FMN and the direct chemical environment in the protein. These observations were supported by the increased lifetimes up to about 6 ns correlating to high signal intensities at 495 nm in our data and the observation, that protein-bound FMN fluorescence increased linearly with a higher glycolytic metabolism in our cohort. Our findings outline that FMN fluorescence plays an important role in metabolic imaging of brain tumors. In the following paragraphs we discuss how FMN fluorescence together with the optical redox ratio contain metabolic characteristics of the brain tumors in our cohort, showing promise for both improved tumor visualization and classification.

### 4.2 Autofluorescence in low- and high-grade gliomas

HGG and in particular glioblastoma show intratumoral heterogeneity with respect to metabolism (40) and glycolytic and oxidative glioma stem cell subpopulations (41). Trinh et al. showed an increase in the fraction of protein-bound NAD(P)H lifetimes in invasive stem-like tumor-initiating cell subpopulations with respect to tumor mass-forming cell subpopulations of malignant glioma (42). This heterogeneity was also reflected by our data, with HGG specimens spanning a rather broad range of optical redox ratios. LGG showed predominantly lower redox ratios and higher protein-bound FMN fluorescence when compared with HGG. Lower optical redox ratios for LGG with respect to HGG were also observed for two-photon excitation at 810 nm (29). This would correspond to a lower fraction of glycolytic activity in HGG compared to LGG. The interpretation of redox ratios, however, is complicated by the fact that glioma cells employ various catabolic pathways for energy production which are linked in multiple ways to anabolic pathways supporting cellular function (43). Biosynthetic demands are increased in cellular differentiation and fatty acid synthesis can further reduce the optical redox ratio (44).

### 4.3 Autofluorescence in meningiomas and metastases

MNG in this study had highly characteristic autofluorescence properties with the overall highest protein-bound FMN fluorescence, highest flavin lifetime and lowest redox ratios among all tumor entities. A strongly increased fraction of aerobic glycolysis in MNG, when compared with glioblastoma and MET, was also found in a study using functional MRI, with about 70% of vital tumor tissue in MNG employing aerobic glycolysis for energy production (45). Considering the linear relation between glycolytic metabolism and increase in protein-bound FMN fluorescence observed in our study, the increased glycolytic activity in MNG is a likely explanation for the high FMN fluorescence and flavin lifetimes. In MET we observed higher redox ratios, which is in accordance with literature (46). A possible explanation could be an acute energy need in contrast to biosynthetic demands for progression as found in other tumor entities (46). Interestingly, flavin lifetimes were still increased in these tumors, providing an additional metric for differentiation from the non-tumorous brain.

### 4.4 Autofluorescence brain tumor classification with machine learning

When correlating the optical redox ratio, *R_flavin_* and the flavin fluorescence lifetime, clusters were observed for the different tumor entities of our cohort. We hypothesized that different tumor entities employ preferential metabolic strategies which are encoded in the tissues’ autofluorescence response and can be used for classification. When fitting our data with a support vector machine, tissue autofluorescence allowed for predicting non-tumorous-brain, LGG and MNG with promising multi-class sensitivity and specificity. However, non-tumorous brain prediction results have to be interpreted with caution due to the low number of patients represented in this study. Prediction was worse for HGG and MET, which becomes obvious when considering the overlap of these groups in **Figure 2**. As in our data, a vast overlap in the rate of aerobic glycolysis for glioblastoma and MET was found in a study employing physiologic MRI (45). HGG were also misclassified as LGG in 21.7% of cases. On the one hand, this can be attributed to the heterogeneous nature of HGG. On the other hand, we hypothesize that this was because histopathological classification is a fluent continuum. For example, a WHO grade II diffuse astrocytoma might already show local characteristics of progression into a WHO grade III anaplastic astrocytoma, which then would lead to the histopathological diagnosis HGG. These differences are harder to resolve when only considering the autofluorescence response.

### 4.5 Limitations

The findings of our study are associated in the light of the following limitations. Our dataset consisted of 108 brain tumor specimens on which 361 data points were collected. While this constituted a rather well representation of various brain tumor entities, robust classification with machine learning models would require more data. In particular, we note that our cohort only included 3 specimens from the non-pathological brain. This is a general limitation of *ex-vivo* studies since specimens from the non-pathological brain are mostly only available during the approach to deep-seated tumors. The optical redox ratio and the fluorescence lifetimes observed in these non-pathological brain specimens corresponded well with literature. Nevertheless, larger studies optimally in the course of multicentre studies are warranted to further increase the dataset. We further note that hemodynamic changes in perfused tissues as the brain can affect intensity-based measurements, such as the optical redox ratio, due to haemoglobin absorption (36). In this study, we selected regions of interest for spectral acquisitions based on fluorescence intensity and lifetime maps and tried to avoid areas with visible blood accumulation. As the flavin lifetime is less affected by changes of the fluorescence intensity and was directly related to the optical redox ratio, it is a promising further biomarker for assessing the metabolic state of tissue. The increase of protein-bound FMN fluorescence with increasing glycolytic activity was underlined by both the fluorescence lifetime and spectra in our work. Nevertheless, follow-up studies should provide additional *in vitro* and *in vivo* analysis to validate our hypothesis. Also, imaging within 1h after resection was very close to *in vivo* conditions. There are a few published studies on the change of autofluorescence signals following resection. Autofluorescence in colonic tissue from NAD(P)H has been reported to decay ex vivo over a time scale of 118 minutes due to tissue deoxygenation, which is about double the time window achieved in our study. Collagen and flavins remained relatively constant (47). The extrapolation of ex vivo findings should always be observed with caution. Nonetheless, the consistency of our results with other studies and imaging systems outline that our ex vivo measurements are a useful first step to investigating the contrast that is likely to be seen *in vivo*. Future studies should also investigate on the complementary value of flavin fluorescence with other established techniques such as protoporphyrin IX fluorescence guidance.

### 4.6 Conclusion and clinical relevance

In the future, the use of flavin fluorescence lifetime and autofluorescence optical redox ratio might be beneficial in supporting the neurosurgeon in visualizing brain tumor tissue during surgery. The following advantages of these techniques are of note: First, this innovative approach might be useful to intraoperatively detect especially LGG tissue that can usually not be visualized by the conventional 5-ALA fluorescence technique. Next, during surgery of meningiomas these techniques might be beneficial in identifying residual tumor tissue especially in neurosurgical sites such as adjacent bone infiltration or the dural tail. Furthermore, potential side effects of other exogenous dyes could be reduced by this new approach. By using these methods enabled by a modified neurosurgical microscope it would be possible to provide a real-time tissue evaluation during resection for improved assessment of the tumor entity and the tumor margin as well as confirmation of diagnostic tumor tissue in stereotactic biopsies.

## Data availability statement

The original contributions presented in the study are included in the article/Supplementary Material. Further inquiries can be directed to the corresponding author.

## Ethics statement

The studies involving human participants were reviewed and approved by Medical University of Vienna (EC no.: 419/2008, amendment). The patients/participants provided their written informed consent to participate in this study.

## Author contributions

GW, BK and LW resected tumor specimens. JG, TR-P and AW performed the histopathological workup. BK, LW, JM and AL organized biopsy handling and preparation. MW and CH provided technical consultation for modifications on the surgical microscope. ME and DR set up the FD-FLIM system under supervision of AU and MA. DR performed the measurements and analysed the data. DR, LW and GW wrote the manuscript. The final version of the manuscript was reviewed and approved by all authors. ME, WD, GW and RL initiated the project. All authors contributed to the article and approved the submitted version.
